# Adhesive Bowel Obstruction (ABO) in a Stranded Narrow-Ridged Finless Porpoise (*Neophocaena asiaeorientalis sunameri*)

**DOI:** 10.3390/ani13243767

**Published:** 2023-12-06

**Authors:** Sung Bin Lee, Adams Hei Long Yuen, Young Min Lee, Sang Wha Kim, Sunmin Kim, Cherry Tsz Ching Poon, Won Joon Jung, Sib Sankar Giri, Sang Guen Kim, Su Jin Jo, Jae Hong Park, Mae Hyun Hwang, Jong-pil Seo, Seongjun Choe, Byung Yeop Kim, Se Chang Park

**Affiliations:** 1 Laboratory of Aquatic Biomedicine, College of Veterinary Medicine, Research Institute for Veterinary Science, Seoul National University, Seoul 08826, Republic of Korea; lsbin1129@snu.ac.kr (S.B.L.); mushhama@snu.ac.kr (Y.M.L.); cwj0125@snu.ac.kr (W.J.J.); ssgiri@snu.ac.kr (S.S.G.); ssjjone@snu.ac.kr (S.J.J.); jaehong139@snu.ac.kr (J.H.P.); ghkdao@snu.ac.kr (M.H.H.); 2 Radiotherapy and Oncology Centre, Gleneagles Hospital Hong Kong, Wong Chuk Hang, Hong Kong SAR, China; yhladams@hotmail.com; 3 Department of Microbiology and Immunology, Institute of Endemic Disease, College of Medicine, Seoul National University, Seoul 03080, Republic of Korea; sangwhakim@kangwon.ac.kr; 4 College of Veterinary Medicine, Institute of Veterinary Science, Kangwon National University, Chuncheon 24341, Republic of Korea; 5 Department of Parasitology, Parasite Research Center, International Parasite Resource Bank, School of Medicine, Chungbuk National University, Cheongju 28644, Republic of Korea; xsunminx@gmail.com (S.K.); vetazmo@gmail.com (S.C.); 6 Department of Surgery, Queen Mary Hospital, Pokfulam, Hong Kong SAR, China; poontccherry@gmail.com; 7 Department of Biological Sciences, Kyonggi University, Suwon 16227, Republic of Korea; imagine0518@kyonggi.ac.kr; 8 College of Veterinary Medicine, Veterinary Medical Research Institute, Jeju National University, Jeju 63243, Republic of Korea; jpseo@jejunu.ac.kr; 9 Department of Marine Industry and Maritime Police, College of Ocean Science, Jeju National University, Jeju 63243, Republic of Korea

**Keywords:** adhesive bowel obstruction, intestinal adhesion, stenosis, diagnostic imaging techniques, narrow-ridged finless porpoise, *Neophocaena asiaeorientalis sunameri*, cetaceans

## Abstract

**Simple Summary:**

This study demonstrates the clinical and radiological presentation of intestinal adhesions and obstructions in a stranded narrow-ridged finless porpoise and discusses the possible cause of death. The main reason for the intestinal lesions was likely related to parasitic infection; however, further studies should be conducted to determine the relationship between parasitic infections and adhesive bowel obstruction. This is the first case study report of intestinal adhesions and obstructions in a narrow-ridged finless porpoise, and it provides a valuable reference for confirming a diagnosis of adhesive bowel obstruction using post-mortem computed tomography (PMCT).

**Abstract:**

In this case report, we present a rare occurrence of a narrow-ridged finless porpoise (*Neophocaena asiaeorientalis sunameri*), discovered on the coast of Jeju Island, Republic of Korea, that was afflicted with adhesive bowel obstruction (ABO), a life-threatening condition that has scarcely been reported in cetaceans. Diagnosis of ABO was confirmed via radiological and clinical assessments. Post-mortem computed tomography and necropsy revealed ABO between two loops of the jejunum at the L8 level. The mesenteric tissue covering the intestinal lesion was severely thickened with increased tension. Both bowel loops were fixed to the mesentery and acutely angulated, leading to asymmetrical thickening of the cross-sectional bowel walls. The intestinal lumen was stenosed because of pressure from the firm mesenteric band, and no fecal matter was observed in the lumen of the posterior bowel or rectum. Calcified nodules were detected, and histological analysis suggested parasitic or suspected post-parasitic infections. The primary cause of the intestinal lesions is presumed to be a reaction related to parasitic infection. However, further investigations would establish a definitive link between parasitic infections and ABO in this species. This case highlights the importance of studying rare medical conditions in wildlife, providing valuable insights into marine mammal health.

## 1. Introduction

Cetaceans have a long gastrointestinal (GI) tract, enabling them to feed on prey of lower caloric value [1], whereas their intestines possess a dependent blood supply from the aorta via the mesenteric artery. However, the long course of the GI tract and the highly mobile mesenteric arterial supply are prone to adhesive bowel obstruction (ABO), which can result in fatal intestinal necrosis and sudden death [2,3].

Unlike domestic animals that often attract greater research attention, ABO is difficult to be detected and measured in wildlife, particularly in cetaceans. Most published records related to ABO in cetaceans were discovered using surgery in captive animals [4] or necropsy in wild animals [5,6]. The study of wildlife health is confronted with numerous impediments, including limitations in the available materials, the behavioral aspects of the animals, as well as considerations in zoology and ecology [7,8]. Consequently, the underlying pathogenesis of this disease in cetaceans is still not fully understood. To seek better understanding of the causes of death and underlying pathology, cetaceans stranded in South Korean waters have been routinely assessed using post-mortem computed tomography (PMCT) to provide additional information complementing the findings of conventional necropsy [9,10]. In this case report, we present a case of ABO in a stranded narrow-ridged finless porpoise and demonstrate the relevant necropsy and PMCT findings.

## 2. Case Description

**Figure 1:**
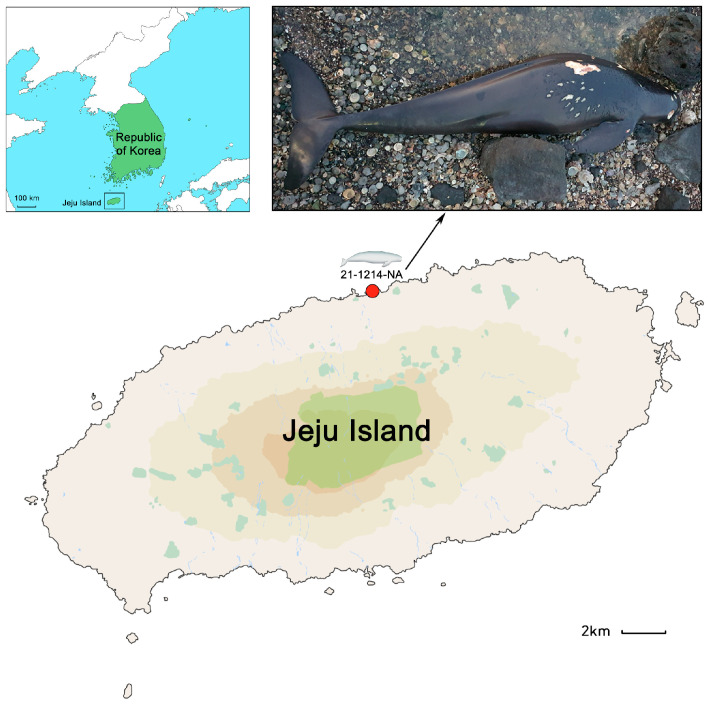
The stranding location of specimen 21-1214-NA. The carcass was discovered on the beach near Hwabukpogu Port Lighthouse in fresh condition (33°52.667′ N 126°56.450′ E).

On 14 December 2021, an adult female narrow-ridged finless porpoise (NRFP) (154.8 cm) was stranded on the northern coast of Jeju Island in the Republic of Korea (33°52.667′ N 126°56.450′ E; Figure 1). The carcass was fresh, and no scavengers were found upon retrieval (code 2) [11]. To minimize post-mortem changes, the carcass was immediately transported to the freezer at the College of Ocean Science (Jeju National University, Jeju-si, Jeju-do, Republic of Korea) upon retrieval, and frozen at −22 °C until further examination. Ethical review and approval were not required because this study did not alter aspects of the animal’s life.

The PMCT was performed using an Aquilion Lightning 16-row, 32-slice helical CT system (Aquilion Lightning, Canon Medical Systems, Otawara, Japan). The scanning parameters were set to 120 kVp and 200 mAs, with a 1 mm slice thickness, and the scanned field of view (sFOV) was 600 mm. The reconstructed PMCT images were assessed using the open-source Digital Imaging and Communications in Medicine (DICOM) viewing software Horos, version 3.3.6 (RRID:SCR_017340). The PMCT multiplanar reconstruction imaging revealed calcified lesions in the gastric wall, rectus abdominis, and left mammary gland, suggesting systemic parasitic infections. 

**Figure 2:**
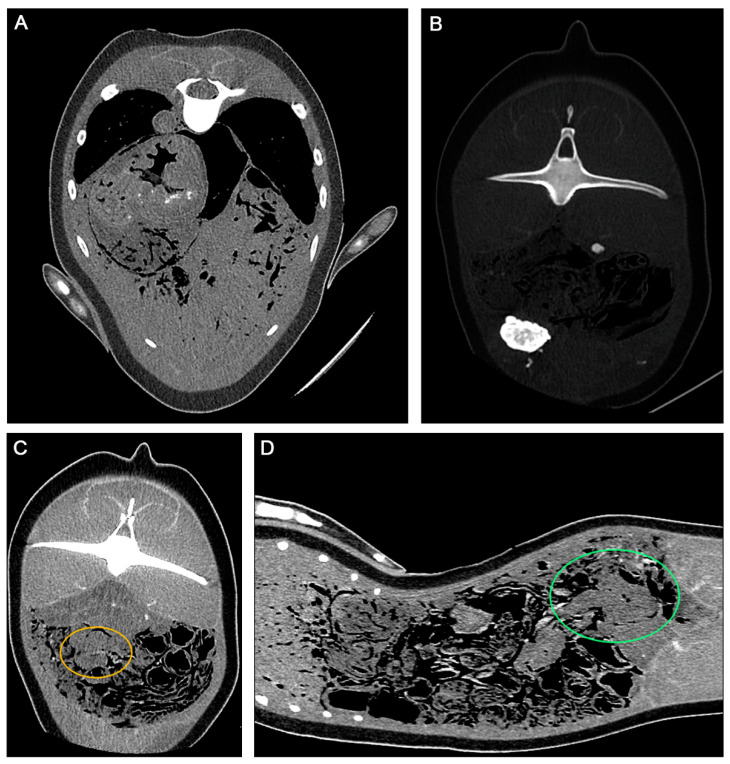
Post-mortem computed tomography (PMCT) images of the individual with suspicion of intestinal adhesion: (**A**) axial PMCT image (WL330/WW830) showing multiple hyperattenuated lesions secondary to parasitic infections. The lesion was an intramuscular nodule near the left mammary gland. (**B**) Using an abdominal PMCT algorithm (WL40/WW350), the forestomach content was observable in a coronal PMCT image, suggesting that the porpoise was actively foraging before death. (**C**) Axial PMCT image (WL40/WW350) showing acute angulation of both bowel loops (orange circle). (**D**) Coronal PMCT image (WL40/WW350) showing mild hyperattenuation in the stenosed intestine, suggestive of fibrotic scar tissue (green circle).

Using a pulmonary image window (WL-500/WW1400), an extensive ground-glass opacity pattern associated with pulmonary infection was observed in both lungs. Gas accumulation was observed in the pleural cavity and pericardium, which led to the suspicion of pneumothorax and pneumopericardium, respectively. The stomach contents were observed, indicating that the porpoise actively foraged before death (Figure 2B); however, the bowel was empty. 

The acute-angled pattern of the intestine (approximately at the L8 level) made up of proximal dilated and distal collapsed bowel loops, with one transition point, was observed (Figure 2C, yellow arrow), which was suggestive of ABO being caused by matted adhesions (WL300/WW3500). Mild hyperattenuation, representing fibrotic scar tissue, was observed at the mesentery where adhesion occurred (Figure 2D) (WL16/WW3500). Radiological findings suggested that the intestinal loop of the carcass may have experienced a resolved intestinal infection. 

Necropsy of the carcass was performed at the Jeju office of the Korea Fisheries Resources Agency (FIRA; Hallim-eup, Jeju, Republic of Korea) on 20 July 2022. During the necropsy of the NRFP, all samples that were suspected to contribute to the cause of death were preserved in 70% ethanol and/or 10% neutral buffered formalin for molecular analysis and histopathological examination, respectively. All the collected samples were transported to the Laboratory of Aquatic Biomedicine (LAB; Seoul National University, Gwanak-gu, Seoul, Republic of Korea), and further analyzed. The formalin-fixed lesions were cut into appropriate sizes, and tissue slides were produced by the Korea Vet Lab (Seongnam, Korea). The samples were embedded into paraffin and sectioned into 5 µm. The sliced tissues were stained using hematoxylin and eosin (H&E), gram, PAS, and Perls Prussian blue to detect histological changes. All tissue slides were analyzed by veterinary pathologists (Antech Diagnostics, Fountain Valley, CA, USA). Parasites were isolated and collected from the tympanic bullae, lungs, liver, gastrointestinal tract, and left mammary glands. Species identification of the parasites was conducted using morphological and genetic analysis at the Parasite Research Center and International Parasite Resource Bank (Chungbuk National University, Seowon-gu, Cheongju, Republic of Korea) [12,13].

Foamy fluid was not observed in the respiratory system. Tension pneumothorax was observed in both lungs, particularly in the left lung. Multiple firm nodules were isolated from the muscles near the left mammary gland (2.9 × 3.7 cm), the outer wall of the forestomach (1.85 × 1.4 cm; 2.3 × 1.1 cm), and the peritoneum (6.0 × 3.1 cm; 4.0 × 3.2 cm) near the genital slit. Based on molecular examination, the nodules near the left mammary gland and peritoneum were identified as *Crassicauda* sp. remains among caseous debris [14]. Although parasitic infection in the forestomach nodules was confirmed in histopathological examination, the species was not identified. The nematode *Anisakis* sp. larvae were identified in the stomachs of the NRFP. The intestine exhibited the presence of *Anisakis* sp. larvae, *Diphyllobothrium fuhrmanni*, and *Synthesium nipponicum*.

**Figure 3:**
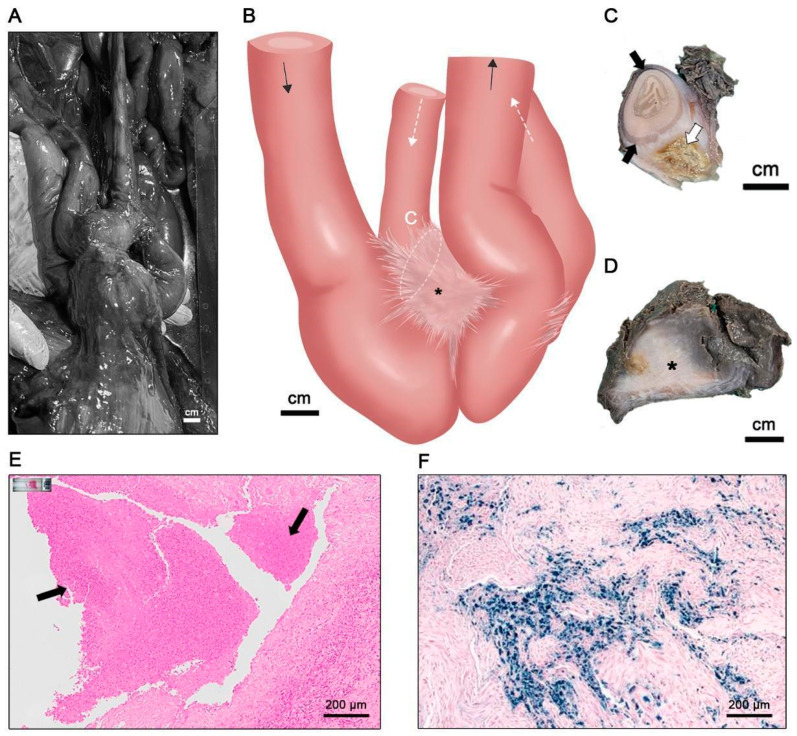
The intestinal lesions of 21-1214-NA. (**A**,**B**) Enteroenteric adhesion was observed between two loops of the anterior and posterior jejunum (**B** was illustrated by S.B.L.). The direction of passage of intraluminal food is indicated by arrows, with black arrows representing the anterior jejunum and white arrows representing the posterior jejunum. The adhesive mesenteric lesion is denoted by an asterisk (*). The loops demonstrated stenosis with (**C**) acute angulation by (**D**) the thickened mesentery band with the adhesive lesion indicated by an asterisk (*). Cross-sectioned intestine loops had asymmetrical thickening of the wall (black arrows in (**C**)) and the mesentery was filled with calcified material (white arrow in (**C**)). Inflammatory cells were centrally infiltrated in the serosa layer of the loops (black arrows in (**E**)). No pathogen was identified in gross examination. (**F**) In the thickened mesentery band, agglomerates of hemosiderin-loaded macrophages were found using Perls Prussian blue staining.

Congestion was observed in the pyloric sphincter of the fundic stomach, which was enlarged. Enteroenteric adhesions were observed between the anterior and posterior jejunum loops (Figure 3A). Two small bowel loops were stenosed (Figure 3B) by the firm mesentery band, which was severely thickened and indurated with increased tension, acute angulation, and asymmetrical wall thickening (Figure 3C,D). The anterior small bowel before the adhesion point demonstrated an enlarged sign, whereas the posterior parts showed normal signs without fecal matter. The transverse sections of the adhered intestines showed inflammation in the serosa layer, which was chronically encapsulated by central inflammatory cells and cellular debris (Figure 3E). No pathogens were identified upon histological examination. In the thickened mesentery band, agglomerates of pigment-loaded macrophages (Figure 3F) were observed. Microabscesses, which are possible traces of migrating parasites [15], were scattered throughout the tissue with mineralization. Finally, no parasites were detected.

## 3. Discussion

In mammals, extensive reports have indicated that ABO is associated with cardinal symptoms such as acute or chronic abdominal pain, vomiting, and decreased oral input [16,17,18]. We believe that these complications may have also affected the NRFP in the present report. The NRFP may have suffered from persistent abdominal pain as a result of ABO. This would have caused the NRFP to regurgitate and vomit whenever it consumed food, and this would be evidenced by fundic gastritis, the presence of stomach content, and the absence of bowel content. Eventually, failure of food passage through the stomach to the bowel would have led to inanition, electrolyte depletion, and consequently, the death of the NRFP [19]. 

Mesenteric fibrosis and stenosis caused by chronic hemorrhage were observed in the reported NRFP, as evidenced by the presence of hemosiderin-loaded macrophages [20]. The etiology of mesenteric fibrosis and stenosis in cetaceans remains incompletely understood, primarily attributed to the scarcity of pertinent disease cases and information. It is hypothesized, however, that localized injury to the mesentery and consequent inflammation may trigger the activation of the fibrinocoagulative pathway [21]. The activated cellular turnover triggers increased infiltration of inflammatory cells and fibrinogen deposition. Consequently, the formation of a fibrous matrix, infiltration of fibroblasts, and consequent fibrin formation lead to permanent adhesion. In the presently reported NRFP, no external scarring was found on the body, indicating mesenteric fibrosis and stenosis resulting from traumatic injury could be ruled out.

The parasitic reaction resulting from *Crassicauda* sp. or *Anisakis* sp. infection could also have been a potential contributing factor to the mesenteric fibrosis and stenosis in this NRFP. This supposition is based on the presence of the parasite within the peritoneum and muscles near the mammary glands, as well as the existence of multiple parasitic or post-parasitic nodules and cysts throughout the body [22,23]. While previous studies have primarily classified *Crassicauda* spp. as endoparasites, they have demonstrated the ability to infect tissues or organs outside their typical habitats [24]. For instance, in Cuvier’s beaked whales (*Ziphius cavirostris*), mesenteric arteritis caused by *Crassicauda* sp. infection led to jejunum–mesentery adhesions [25]. In contrast, prior studies have reported instances of small bowel obstruction in humans resulting from acute enteric anisakiasis following the consumption of raw seafood [26,27]. Enteric anisakiasis is a rare occurrence, accounting for 4.1% of human anisakiasis cases [28], and the symptoms observed in these human cases align with those identified in the NRFP of this study, including focal stenosis, wall thickening, and inflammatory changes in the jejunum. Although direct parasitic invasion of the bowel wall was not observed in the intestinal lesions of the NRFP, the occurrence of ABO could be attributed to an acute anaphylactic allergic reaction [29], given the presence of *Anisakis* sp. larvae in the intestine, a rarity in this species as well. However, further investigations are necessary to establish a causal link between intestinal adhesions, parasitic infections, and the life cycle of *Crassicauda* spp. and *Anisakis* spp. in narrow-ridged finless porpoises (*N. asiaeorientalis sunameri*).

In recent years, active research has been conducted on the indirect assessment of wildlife disease processes in free-ranging wild animals using various techniques such as satellite remote sensing, camera traps, and echolocation signals [30,31,32]. However, the underwater environment presents physical and technical challenges such as limited visibility, underwater acoustic sensor networks, and wireless equipment setup difficulties [33,34,35]. Particularly, in the case of finless porpoises, the absence of dorsal fins and their inconspicuous swimming behavior, scarcely exhibiting aerial body parts, pose significant limitations in disease monitoring, contributing to a slower pace of research compared to terrestrial animal disease assessments [36]. Due to these limitations, we are conducting disease-monitoring examinations using cetacean carcasses. Specifically, post-mortem computed tomography (PMCT) has emerged as a standard initial screening procedure for cetaceans in Korean waters. It provides non-invasive assessment and strong evidence to demonstrate various pathological conditions in cetaceans. The datasets obtained from PMCT can be easily stored digitally, allowing requests for a second opinion or retrospective examination. However, the diagnosis of ABO using PMCT is highly dependent on the operator. The best practice for diagnosing ABO is using CT scanning with contrast media to enhance the density differences between lesions and the surrounding parenchyma. Contrast-enhanced post-mortem computed tomography requires a roller pump to allow good perfusion of the blood vessels and to ease the visibility of ABO. Unfortunately, the imaging center does not currently have a roller pump, making the application of contrast media impossible in this case. Therefore, the operators must be familiar with the anatomy of NRFPs, as well as the use of PMCT reconstruction techniques, to accurately locate the affected site. In this study, PMCT procedures and image evaluations were performed under the guidance of a board-certified radiographer (A.H.L.Y.) with over 7 years of experience in post-mortem cetacean imaging. It is crucial that operators are accurately trained to implement the diagnostic capabilities of computed tomography to diagnose ABO in cetaceans.

To the best of our knowledge, the published literature lacks any reference to the applicability of imaging modalities in diagnosing intestinal adhesion and stenosis in NRFPs. Computed tomography is a frequently used modality for identifying intestinal adhesions in humans. It can be used to track the level of intestinal adhesion by identifying the “whirl” sign, the fat notch sign, the beak sign, and the small bowel feces sign [37]. Together with the cardinal symptoms of intestinal obstruction such as abdominal pain, nausea or vomiting, and absolute constipation, diagnosis of intestinal adhesions in living organisms can be much more straightforward. However, in the case of a dead organism, obtaining information regarding clinical symptoms becomes impossible. In the present study, our group adopted the PMCT workflow and imaging parameters with reference to Yuen et al. [9,10] for the diagnosis of abdominal gas embolism and upper aerodigestive tract obstruction. We found that mesenteric fibrosis was a significant radiological sign to propose the presence of intestinal adhesion, which was confirmed via necropsy. In the future, research should be directed at obtaining a standardized CT imaging sign for the diagnosis of intestinal adhesion.

Cetaceans serve as umbrella species at the top of the marine ecosystem [38,39], playing a crucial role as keystone species that define the entire environmental conditions [40], including various factors such as micro-plastics, contaminants, epidemics, and pollution [41,42]. However, the NRFP is currently listed as an endangered species on the International Union for Conservation of Nature (IUCN) Red List, reflecting a significant decline in its population in recent years [43]. To address this issue, it is imperative to establish a continuous monitoring system for the NRFP population and the emergence of diseases within the marine environment of Korean seawater. This case report aims to contribute to further veterinary studies of NRFPs by providing insights into the pathology and living conditions of NRFPs in the Korean sea.

## 4. Conclusions

This case report documents ABO in an NRFP, and it uses PMCT as an adjunct to confirm this fatal condition, which we diagnosed via necropsy. PMCT has emerged as a standard initial screening procedure and non-invasive assessment for cetaceans in Korean seawaters. The findings highlight the merit of utilizing CT for the identification of ABO in cetaceans; therefore, this report will serve as a reference for the diagnosis of ABO in live or dead cetaceans. The etiology of this ABO case is postulated to involve parasitic infection, akin to instances in humans; however, this remains unclear, necessitating further investigation. The health and pathology of the population of NRFPs in Korean seawater, classified as an endangered species, should undergo continuous monitoring. We believe that this study can offer insights for further comprehensive investigations.

## Data Availability

Substantial research data from this study have been presented in the paper. Access to original data is available on request.
